# Anticonvulsant Effects of Dingxian Pill in Pentylenetetrazol-Kindled Rats

**DOI:** 10.1155/2019/4534167

**Published:** 2019-03-18

**Authors:** Yudan Zhu, Shuzhang Zhang, Mei Shen, Zhiping Zhang, Kan Xu, Jiwei Cheng, Yiqin Ge, Jie Tao

**Affiliations:** ^1^Central Laboratory, Department of Neurology and Neurosurgery, Putuo Hospital, Shanghai University of Traditional Chinese Medicine, Shanghai, China; ^2^Laboratory of Neuropharmacology and Neurotoxicology, School of Life Science, Shanghai University, Shanghai, China

## Abstract

Dingxian pill has been used as an antiepilepsy agent in China from ancient to modern times, of which the concrete pharmacological characterization and the underlying molecular mechanism remain unclear. The present study was undertaken to investigate them by animal behavior, electroencephalogram (EEG), Morris water maze, immunohistochemistry, transcriptomics, and real-time PCR. In our results, the treatment of Dingxian pill dose-dependently inhibited PTZ-induced seizure-like behavior and reduced the seizure grades, LFP power spectral density, and brain wave of the epileptiform EEG component induced by PTZ. In Morris water maze tests, the learning and memory ability of kindled epileptic rats could be attenuated more efficiently by Dingxian pill. For the immediate early gene c-fos, the expression was reduced after Dingxian pill treatment, and the difference was significant between the treatment and the model group. Through the transcriptome analysis of the gene expression in hippocampus, Egr3, Nrg, Arc, and Ptgs2, closely related to epilepsy, had been proved to be downregulated by application of Dingxian pill. All of the results not only highlight the antiepileptic effects of Dingxian pill and its molecular mechanism, but also provide a modern validity theory for the clinical application of traditional Chinese medicine (TCM).

## 1. Introduction

Epilepsy, as one of the most common and treatable neurologic diseases, is invoked by the abnormal discharge of brain neurons and characterized as a progressive loss of neurological function [1]. So far, 9 millions of people have suffered epilepsy in China, which is about one-sixth of the world's population suffering from epilepsy, and the sufferers were grown with 300-400 thousands in every year [2, 3]. Moreover, sudden unexpected deaths in epilepsy (SUDEP) is an important factor of premature death [4]. On the one hand, the routine antiepileptic drugs focus on reducing the convulsive symptoms with side effects such as cognitive impairment or liver injury [5–8]. On the other hand, 30% of patients, without being improved the pathogenesis after treatment with conventional antiepileptic drugs, still develop into the intractable epilepsy [9–12]. Therefore, it is urgent to explore effective drugs and pharmacological mechanisms for the treatment of epilepsy.

Dingxian pill has been widely used in treating epilepsy, as a classic prescription for treating epilepsy in China, containing Gastrodia elata, Scorpion, Bombyx batryticatus, Tendrilleaf fritillary bulb, Ternate pinellia, Indian buead, Bile Arisaema, Acorus gramineus, Amber, Tangerine peel, Thinleaf milkwort root, salvia miltiorrhiza, Dwarf lilyturf root tuber, Mercury blende, and bamboo juice. The application of Dingxian pill is extended to treat the temporal lobe epilepsy and pediatric epilepsy. Compared with the clinical efficacy of routine antiepileptic drugs in pediatric epilepsy, the total effective rate of Dingxian pill (87.5%) was higher than that of sodium valproate group (75%,* P*<0.05) [13]. In foundational research, Dingxian pill had a significant effective effect for temporal lobe epilepsy by reducing the frequency of seizures in rats [14]. It not only prevents the seizure of rats, but also prolongs the incubation period of convulsions on penicillin, an antagonist to GABA receptors, induced epilepsy rats [14]. Meanwhile, the glutamate content decreased, GABA level increased, and the expression of c-fos was suppressed by Dingxian pill in the hippocampus of rats [15]. The results partly implied the anticonvulsant effects of Dingxian pill; however, the mechanism is still unclear.

So far, as the epilepsy has been a hot issue, the targets such as voltage-gated sodium channels and GABA receptors on the neuron have attracted more and more attention. Additionally, c-fos is one of immediate-early gene and has a strong connection between the voltage-gated sodium channels and GABA receptor [12, 16]. Thus more attention and study have paid attention to a suitable traditional herbal Chinese medicine of seizure, which could improve the therapeutic effect and reduce the side effects in digestive, nerves, and cognition function system [17]. From the Dingxian pill, Gastrodia elata, which was an herbal medicine, has been shown to have remarkable anticonvulsant effects on various rodent models of epilepsy in vivo [18], and its active ingredient Gastrodin (GAS) could decrease seizure severity and recovery time through inhibiting Nav1.6 sodium currents in a gerbil epilepsy model [19]. Scorpion could significantly reduce the incidence and average duration of convulsion in rats, and the sodium channels were inhibited by the bioactive extract BmK IT2 and BmK AS [20, 21]. Bombyx batryticatus has great treatment effects on the central nervous system disease, including antiepileptic, anticonvulsant, hypnotic effects, and so on. Although the latent period of isoniazid-induced or nikethamide-induced convulsion in mice could be prolonged by the beauvericin, the mechanisms of biological activities of Bombyx batryticatus should need to be further explored [22]. These studies of components also partly reminded the antiepileptic mechanism of Dingxian pill.

The previous studies of Dingxian pill are mainly aimed at the intervention effects and molecular mechanism of single herb or single agent rather than the prescription on epilepsy. In the present study, the behavior tests, EEG recording, and water maze are performed, and the effect of Dingxian pill on c-fos expression was also studied. Finally the possible molecular mechanism underlying the antiepileptic effects of Dingxian pill was illustrated by transcriptomic analysis.

## 2. Materials and Methods

### 2.1. Reagents

Dingxian pill was prepared in accordance with standards formulated from the Jiangsu Hospital of Traditional Chinese Medicine (Nanjing, Jiangsu Province, China). Pentrazol (PTZ) and pentobarbital sodium salt were purchased from Sigma Aldrich Co. (St. Louis, MO, USA), sodium valproate (VPA) from Sanofi-Aventis Co. (Paris, France), and anti-c-Fos from Novus Biologicals (NB110-75039, Colorado, USA). The SABC-POD kit, DAB coloring kit, and IgG-Biotin were obtained from Boster Company (Wuhan, China). The kits of RT-PCR and Trizol were purchased from Novizan Biotechnology Co. (Nanjing, China).

### 2.2. Animals

The number of 70 male Sprague-Dawley rats (body weight 200-220 g) was obtained from Shanghai Slac Laboratory Animal Co. Ltd. (Shanghai, China). They raised under controlled conditions at temperature (25±2°C) and 12h light-dark cycle with free access to food and water, for at least seven days before experiment. All experiments performed in animals were in accordance with the China legislation on the use and care of laboratory animals and approved by the Animal Care and Use Committee of Shanghai University of Traditional Chinese Medicine (ACSHU-2011-G115).

### 2.3. Establishment of Epilepsy Rat Model and Drug Treatment

Pentylenetetrazol (PTZ) water solution (35 mg/kg) was administered by intraperitoneal injection for 28 days to induce chronic epilepsy model. On the fifth day after operation, the PTZ solution (35 mg/kg) was injected into rats again, and within 1 hour following the injection, the rats started to develop symptoms of seizures. The seizure activities were rated according to the Racine Scale by observing behavioral postures (i.e., lordosis, straight tail, jumping/running, forelimb clonus, and/or rearing) [23]. Then animals that exhibited at least 2 recurrent seizures per day were selected for further experimentation [24]. At the end of the experiment, 49 rats were divided into 5 groups for further experimentation.

Following the PTZ injection (i.p.), the 5 groups of rats were treated with different drugs once a day for 28 days. In detail, the solutions of Dingxian pill were applied intragastrically to 3 groups (low, middle, and high) at a dose of 0.6 g/kg, 1.2 g/kg, and 2.4 g/kg, respectively. The remaining two groups were treated with VPA (0.2 g/kg) and the same volume of saline. During the treatment period, rats were injected with PTZ again in the 7th, 14th, 21st, and 28th day.

### 2.4. Electrophysiological Recordings of Rats

The rats were fixed in the stereotaxic instrument under anesthesia with pentobarbital in a dose of 30 mg/kg (i.p., 2.5%). When the rats were deeply anesthetized, making a 20 mm incision on the head using a scalpel, then find the bregma and lambda points on the skull by pulling the scalp away with forceps. The pacing electrodes were applied to the sites (AP: 4.0 mm, L: 2.2 mm; H: 2.5 mm) through consulting the Rat Brain in Stereotaxic Coordinates [25], and the rats were subjected to craniotomy with dentist's micro-drill. The dura was broken with a syringe needle, and the depth of the electrode should be inserted into 2.2±0.2 mm below the hippocampus CA1. After full recovery, connect the electrodes implanted on the skull of rats to the amplifier in its own cage. Connect the amplifier to an analogue-to-digital converter and attach the converter to a computer. After getting the baseline recording, inject the pup intraperitoneally with PTZ (35 mg/kg) to induce epileptic seizures. 1 hour after the PTZ injection observe and record the epileptic discharges.

### 2.5. Morris Water Maze Test

The detailed procedure of Morris water maze (MWM) test was conducted according to the described previously [26]. Forty-nine epilepsy rats were randomly divided into 5 groups (n=9-10/group), namely, vehicle-treated group, VPA (0.2 g/kg)-treated group, low dose of Dingxian pill (0.6 g/kg)-treated group, middle dose of Dingxian pill (1.2 g/kg)-treated group, and high dose of Dingxian pill (2.4 g/kg)-treated group. Dingxian pill and VPA were administered by oral gavage for 4 weeks prior to the behavioral testing. Then in the testing, the escape latency and path length were recorded and analyzed.

### 2.6. Immunohistochemistry

All rats were anesthetized with PTZ (35 mg/kg) 2 h after injection and were perfused transcardially with 50 mL of saline followed by 200 mL of 4% paraformaldehyde. Brains were removed and 20 *μ*m coronal frozen sections cut on a sliding knife microtome. The sections were collected in a cryoprotective solution. Immunohistochemical staining for c-fos was carried out via the avidin-biotin procedure. Briefly, the sections were first incubated for 30 min in 10% normal serum plus 5% BSA in PBS to block nonspecific binding and then were incubated overnight at 4°C with rabbit polyclonal c-fos antiserum (1:500 dilution). For control purposes, the sections were either incubated with antiserum that had been exposed to saturating levels of the c-fos peptide (preabsorption control) or incubated in the absence of the primary antibody. The sections were then incubated with an anti-rabbit biotinylated IgG for 45 min and subsequently reacted with SABC for 20 min. The peroxidase reaction was developed in a chromagen solution containing 100 mM nickel sulfate, 125 mM sodium acetate, 10 mM imidazole, 0.03% diaminobenzidine (DAB), and 0.01% hydrogen peroxide at pH 6.5. The sections were then mounted and photomicrographed [27].

### 2.7. Quantitative RT-PCR Analysis

Total RNA was prepared from rat brain hippocampus, and mRNA levels of Egr3, Nrg1, Arc, and cox-2 were measured using the SYBR Green PCR Master Mix Kit (Nanjing, China) on a 7500 FAST Real-Time PCR System (Applied Biosystems, Foster City, CA, USA) with the GADPH as an internal control.

### 2.8. Statistical Analysis

All data were analyzed using the Origin 8.5 (OriginLab, USA) and expressed as mean±SEM unless otherwise indicated. The escape latency and escape rate data in MWM test were analyzed using one-way analysis of variance (ANOVA) with repeated measures. The other behavioral data and the biomarkers changes were analyzed by one-way ANOVA followed by Tukey's post hoc test. For all statistical tests, the value of* P* < 0.05 was regarded as significant.

## 3. Results

### 3.1. Anticonvulsant Effects of Dingxian Pill on Seizure-Like Behavior Induced by PTZ

**Figure 1:**
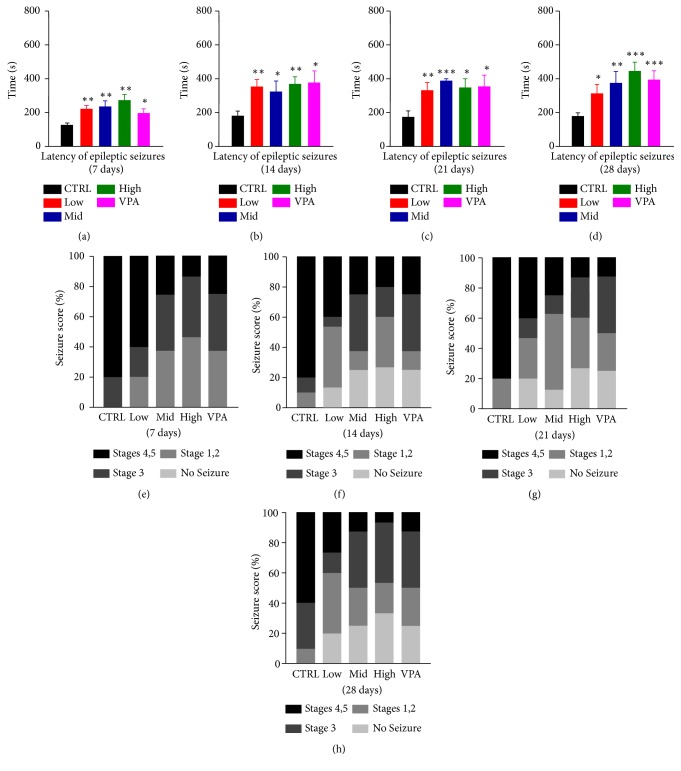
The anticonvulsant effects of Dingxian pill on seizure-like behaviour induced by PTZ: the latency of epileptic seizures ((a)–(d)) and the percentage of seizure stage ((e)–(h)).

The stereotypical oral and masticatory movements, hypokinesia, head bobbing, and wet-dog shakes were developed, following the systemic administration of PTZ (35 mg/kg). And the initial behavior rapidly progressed through the kindling stages from the seventh day. To explore whether Dingxian pill could prevent against PTZ-induced chronic epilepsy, the latency of seizure among the groups was observed. As Figure 1 showed, the latency of high dose group was increased remarkably compared to the control rats in the 14th, 21st, and 28th day (Figures 1(b)–1(d), 368.47 ± 43.63 s versus 183.00 ± 26.87 s* P*< 0.05; 347.19 ± 51.94 s versus 173.20 ± 38.28 s* P*< 0.05; and 445.06 ± 52.33 s versus 189.70 ± 24.78 s* P*< 0.001) and the high dose group had no significant difference with VPA group (Figure 1(d) 445.06 ± 52.33 s versus 394.38 ± 53.67 s) in the 28th day. Besides, the incubation period of the middle dose group was significantly longer than that of the CTRL group at day 21 (Figure 1(c) 347.18 ± 51.94 s versus 173.20 ± 38.28 s,* P*< 0.001).

For the percentage of seizure stage, the Dingxian pill treatment groups showed a significant decrease than control group in seizure 4,5 from the 7th day, especial for the high group ( 13.33% versus 80%, 7th day; 20% versus 80%, 14th day; 13.3% versus 80%, 21st day and 6.67% versus 60%, 28th day, Figures 1(e)–1(h)).

### 3.2. Modulatory Effects of Dingxian Pill on the Electrographic Seizures Induced by PTZ

**Figure 2:**
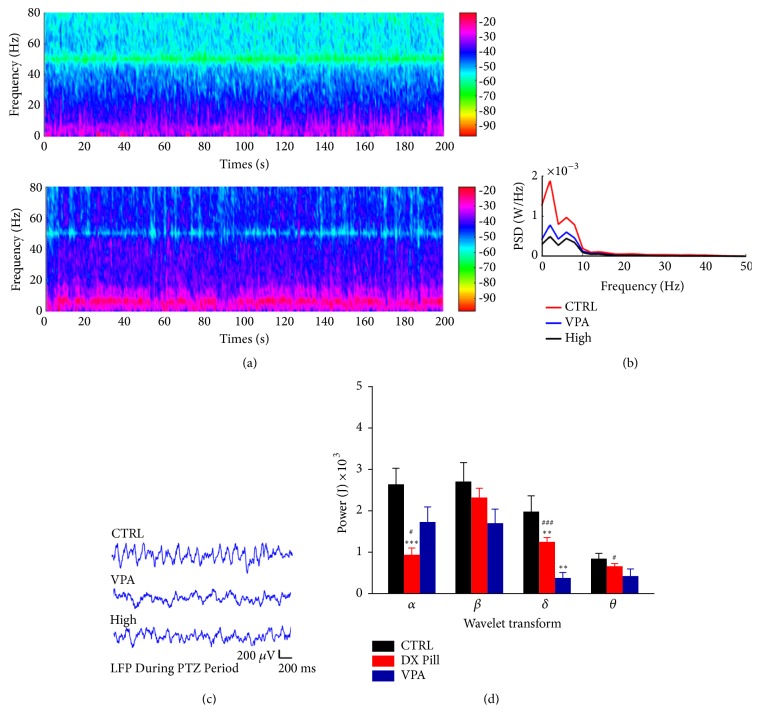
The effect of Dingxian pill on the electroencephalogram (EEG) in rats with chronic preeclampsia induced by PTZ: the EEG spectrum analysis of high dose group ((a) figure above) and control group ((a) figure below). The average power spectral densities (PSD) of all the frequencies among CTRL, high, and VPA group (b). The representative local field potential (LFP) of epilepsy rats recorded during PTZ period (c). The energy of each single waveform in rats during PTZ period among CTRL, Dingxian pill, and VPA group (d).

To explore whether Dingxian pill could affect the level of epileptiform EEG traces, the VPA and the high dose of Dingxian pill (2.4 g/kg) treatment groups were analyzed after PTZ treatment. As shown in Figure 2(a), the spectrums of control and high dose of Dingxian pill treatment groups were compared.

An increased seizure frequency was found in both groups, but the frequency of control group was increased compared to the high dose group significantly after PTZ. The comparison of the mean power spectral density among the CTRL, VPA, and Dingxian pill groups showed sharp decrease of spectral power around the CTRL group (Figure 2(b)).

While the LFP of the three groups was enhanced in the hippocampus following the PTZ induced, the amplitude of the local field potential in the high dose group and the VPA group was lower than that in the CTRL group (Figure 2(c)).

Based on deep study, which is shown in Figure 2(d), there was a general tendency that the activities of brain waves in the high dose of Dingxian pill and VPA groups were decreased, and the decrease of Dingxian pill reached statistical significance in the brain wave *α*, *δ*, *θ* (0.93 ± 0.17 versus 2.64 ± 0.38,* P*< 0.001; 1.25 ± 0.11 versus 1.98 ± 0.38,* P*< 0.01; 0.66 ± 0.06 versus 0.85 ± 0.13,* P*< 0.05, respectively), compared to the CTRL group.

### 3.3. Dingxian Pill Attenuated Cognitive Impairments of PTZ Induced-Epileptic Rats

**Figure 3:**
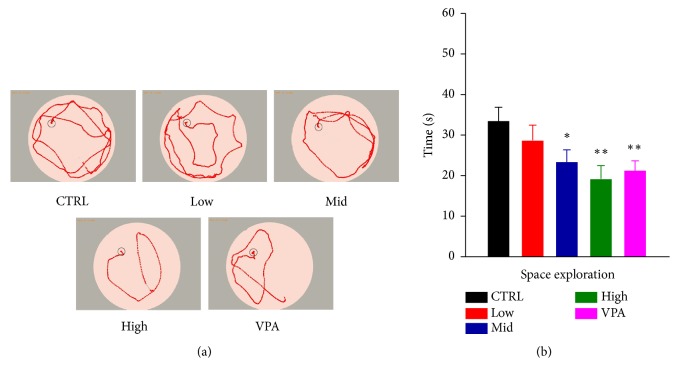
Effects of Dingxian pill on PTZ-induced memory-impaired rats: swimming tracks of rats in water tank (a) and escape latency of rats in hidden platform tests on the fifth testing day.

The improvement of memory and cognition in the treatment groups was measured in PTZ-induced epilepsy rats by the Morris water maze (MWM) tasks. In the hidden platform-swimming trials, the mice of VPA and high dose of Dingxian pill-treated groups (except the low and middle dose) showed markedly improving memory as the path length (Figure 3(a)) and their escape latency (Figure 3(b), 21.13 ± 2.50 versus 33.31 ± 3.49,* P*<0.01; 19.06 ± 3.37 versus 33.31 ± 3.49,* P*<0.01) on the fifth testing day were effectively shortened compared to the control. Above all, both VPA and the high dose of Dingxian pill administration significantly improved spatial learning and memory of cognitively impaired mice.

### 3.4. The Expression of c-Fos Was Decreased by Dingxian Pill in the Hippocampus of PTZ Induced-Epileptic Rats

**Figure 4:**
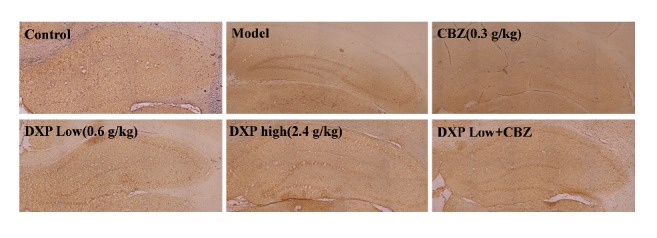
The expression of c-fos in the hippocampus cortex of epilepsy rat among control, model, VPA, DXP-low, DXP-middle, and DXP-high groups.

To explore the effect of Dingxian pill on the expression of immediately early gene c-fos in PTZ kindling epilepsy rat brain, the protein expression was compared in the different treatment groups by immunohistochemistry assay, exhibited in Figure 4. The expression of c-fos positive cells in the high dose group (*P*<0.01) and VPA (*P*<0.05) group was significantly lower than that in the CTRL group and the low dose group. These results indicated that Dingxian pill significantly antagonizes the c-fos protein expression of rat hippocampus caused by PTZ, which may be one of the mechanisms of Dingxian pill prevention and treatment of epilepsy.

### 3.5. The Transcriptomic Analysis of Dingxian Pill Treatment on PTZ Induced-Epileptic Model

**Figure 5:**
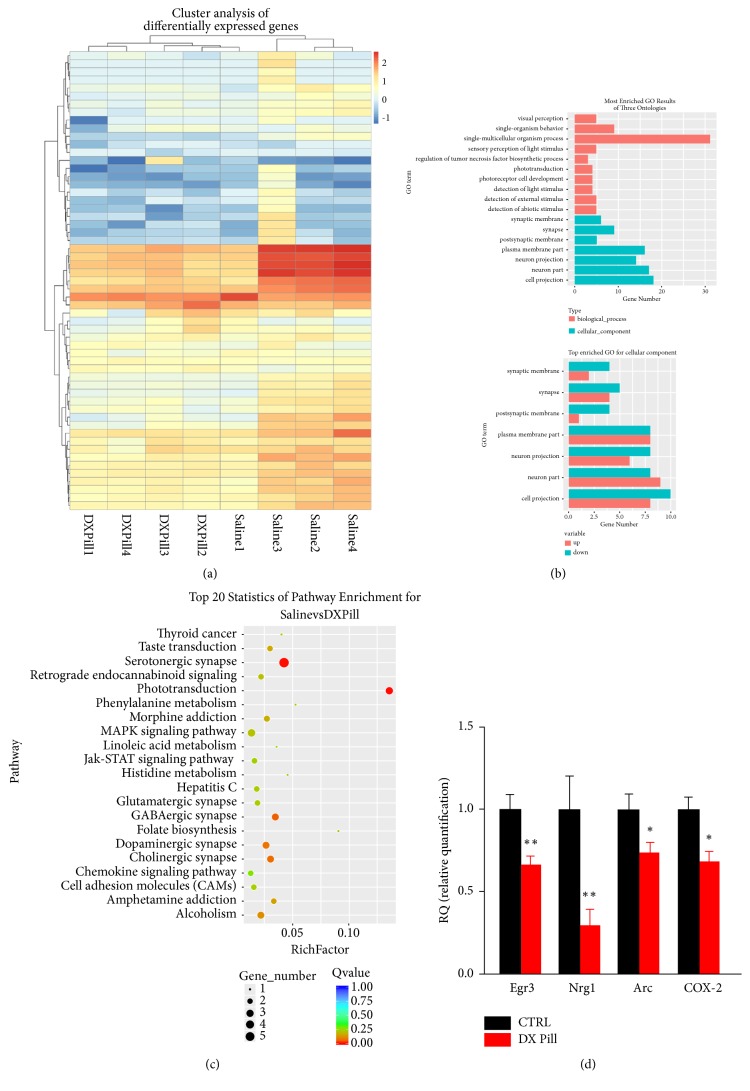
The transcriptome analysis of Dingxian pill-intervened rats: analysis of differentially expressed genes ((a), each column represents an experimental condition, each row represents a log2 ratio value of a gene or log10 (FPKM + 0.01), and different expression variants or expression levels are expressed in different colors.); the analysis of GO function of differentially expressed genes (b); KEGG pathway enrichment analysis of differential expression genes (c); and the comparison of differential gene expression between CTRL and DX pill group by real-time PCR analysis (d).

Molecular mechanism of Dingxian pill intervention in chronic epilepsy had been explored by the transcript analysis (Figure 5). The results indicated that there are 57 differentially expressed genes in the intervention group and the control group (more than 2 times the expression level), some of which are closely related to epilepsy such as Egr3, Nrg1, Arc, and cox-2. Based on GO and pathway analysis, differentially expressed genes are mainly involved in the function of synaptic plasticity, receptor binding, redox and neurotransmitter secretion, and signaling pathway for MAPK-ERK, JAK-Stat, and GABA receptor synthesis. In addition, quantitative RT-PCR analysis showed that the mRNA level of Egr3 (0.66±0.05), Arc (0.74±0.06), and cox-2 (0.68±0.06) was obviously decreased compared with control group (1.00±0.09) (*P*<0.01,* P*<0.05,* P*<0.05, n=5, respectively), especially for Nrg1 (0.30±0.09,* P*<0.01, n=5).

## 4. Discussion

Epilepsy is a common neurodegenerative disease, and the voltage-gated sodium channels, GABA receptor, and c-fos are closely related. As a hot and difficult problem in modern time, the effective and few side-effective drugs are still to be discovered, especially for the temporal lobe epilepsy. Dingxian pill is used for treating epilepsy in China, but the mechanism is still unclear. Thus, the pharmacodynamics and the mechanism of Dingxian pill were researched in present study.

The results showed that long-time Dingxian pill treatment could reduce the seizures frequency through inhibiting the abnormal discharge of hippocampal neurons and the expression of c-fos gene, which is closely related to epilepsy. It shows that c-fos is commonly present in the central nervous system and the expression level of c-fos is generally low, but the expression would be increased with epileptogenesis [27]. Gastrodia elata, which is one component of Dingxian pill, could antagonize the impairment of learning and memory on c-fos expression [28]. BmK IT2, a *β*-like neurotoxin containing 61 amino acid residues, was extracted from scorpion, a Chinese drug in Dingxian pill. Application of different doses of BmK IT2 (0.05, 0.1, and 0.5 mg) induced a dose-dependent suppression of the c-Fos expression in hippocampus [29]. Dingxian pill also could be improving the learning and memory on memory deficits in chronic epilepsy rats. In addition, the significant effect of Dingxian pill may be via mediating the Egr3-GABRA4, NRG1-ErbB4, MAPK-ERK-Arc, and cox-2-Pgp signal-pathways, which were associated with epilepsy. For the Egr3-GABRA4 pathway, Egr3 as a critical regulator of endogenous GABRA4, one of the inhibitory subunits underlying the GABA_A_ receptor, during development, had a major role in developing neurons and in epileptogenesis [28, 29]. Even more interesting, the mRNA expression of GABRA1-4 had been compared in our study. The results showed that the mRNA level of GABRA1 after Dingxian pill treatment was elevated compared with PTZ group, but the mRNA level of GABRA4 was decreased and others had no change, which indicated that antiepileptic effects of Dingxian pill may be closely related to GABRA1 and GABRA4 (Supplementary Material, GABRA1, saline: 1.00±0.22, Dingxian pill: 1.97±0.29; GABRA4, saline: 1.00±0.17, Dingxian pill: 0.54±0.04, n=3, P<0.05, SFigure1). As for NRG1-ErbB4 pathway, downregulation of NRG1 expression could improve the activity of voltage-gated sodium channel and the excitability of ErbB4 positive intermediate neurons, then promoting the release of GABA, inhibiting the discharge of hippocampal pyramidal neurons, and interfering with the occurrence of epilepsy [30, 31]. The activity-regulated cytoskeletal associated protein (Arc), as a marker neuronal activation, was associated with the granule cells born after pilocarpine-induced SE [32], and the expression of Arc was reduced after therapeutic interventions [33]. In addition, the P-glycoprotein (Pgp) was overexpressed after seizure and the mechanism with glutamate could enhance the Pgp expression in the blood-brain barrier through NMDA receptor and cox-2 [34].

The temporal lobe epilepsy (TLE) as a research focus, the hippocampal sclerosis, is the most common neuropathologic finding, including neuron loss, glial proliferation, and synapse formation [26]. Although new antiepileptic drugs have been introduced into clinical practice, their effects remain poor [35, 36]. The pathogenesis of TLE is complex and still unclear, but the possible mechanisms of resistance were concluded, including drugs hard to reach the target, the targets changed, and the true target unknown. Thus exploring and developing new antiepileptic drugs are of great significance for further study.

So far, the treatment of TLE by Chinese traditional medicine has a better curative effect on inhibiting the frequency of epilepsy, improving curative effect, and reducing side effect of clinical treatment. Many studies have showed that the traditional Chinese medicine (TCM) has effective clinical effect on treating TLE. And it looks like patients would be safer on TCM than on other drugs for long periods of time. Thus TCM would be the best choice for the patients, when considering the fear of attacks, concerns about long-term medication.

The traditional Chinese medicine has a centuries-old tradition of use by epilepsy patients around the world. And herbal therapies are often used for general health maintenance or for chronic conditions like epilepsy in developing countries [37]. In addition, clinical experience suggests that patients may try some herbal therapies in order to reduce AED-related adverse effects or comorbid conditions [38]. Thus, many Chinese herbal prescriptions, Chinese herbs, and the monomer of treating epilepsy were compared by the modern molecular biotechnology. For the antiepileptic herb, Gastrodia elata blume (GE) is used to treat epilepsy in East Asia, which is one medicine of Dingxian pill. And gastrodin, a phenolic glucoside derived from GE, had been the major monomer for antiepilepsy [39]. It is indicated that herbal prescriptions, herbs, and monomer have been shown to have neuroprotective properties [40–42], efficacy in animal models of epilepsy [43–45] and hippocampal slice models [46], and effects on gene expression [47]. However, the effective basic principles of many herbal prescriptions have not been studied, because the ingredients are complicated. Through the “effective on the basis of chemical substances” study, the efficacy of Chinese medicine would be clarified.

## 5. Conclusion

Dingxian pills can relieve the seizures of chronic epilepsy rats injected by PTZ through inhibiting the abnormal discharge of hippocampal neurons in epileptic rats. The treatment of epilepsy pill can effectively improve the spatial learning and memory ability of epilepsy model. The Dingxian pills might interfere with epilepsy as well as cognitive dysfunction through Egr3-GABRA4 signal, NRG1-ErbB4 signal, and ERK-Arc pathway.

## Data Availability

The data used to support the findings of this study are included within the article.

## Supplementary Materials

Supplementary Materials Supplementary Figure 1: effects of DX pill on the expression of GABAA receptor subunits in PTZ-induced seizure model as detected using real-time PCR. Data are expressed as the mean±S.E.M., n=3, P>0.05, compared with the saline group.
