# Supplementary material for: Anticonvulsant Effects of Dingxian Pill in Pentylenetetrazol-Kindled Rats
Source: Evid Based Complement Alternat Med. 2019 Mar 18;2019:4534167. doi: 10.1155/2019/4534167 (PMC6442303; doi:10.1155/2019/4534167)
Supplement: Supplementary Materials — Supplementary Figure 1: effects of DX pill on the expression of GABAA receptor subunits in PTZ-induced seizure model as detected using real-time PCR. Data are expressed as the mean±S.E.M., n=3, P>0.05, compared with the saline group. [file 4534167.f1.docx]

**Supplementary Material**


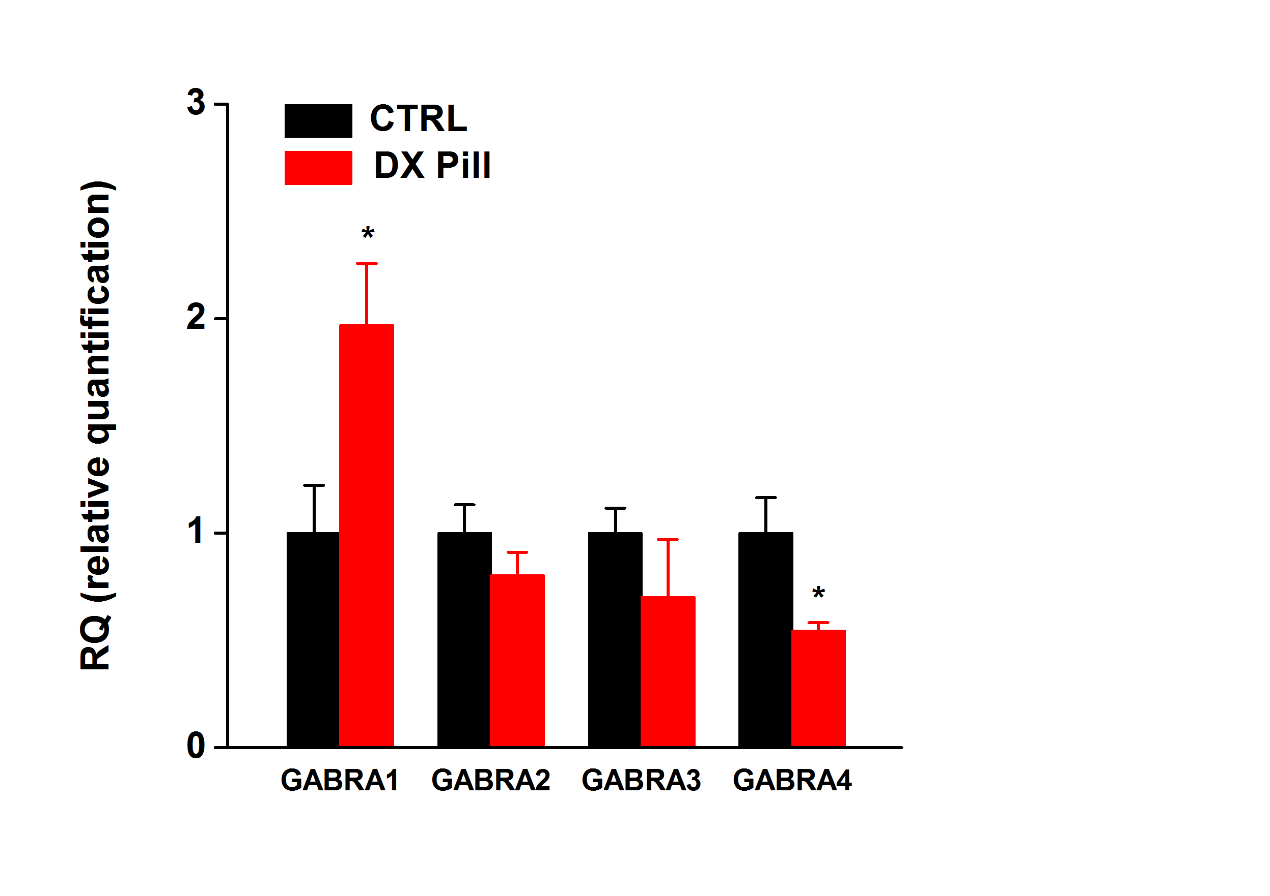


**Supplementary Figure 1** Effects of DX pill on the expression of GABA_A_ receptor subunits in PTZ-induced seizure model as detected using Realtime PCR. Data are expressed as the mean±S.E.M., n=3, P>0.05, compared with the saline group.
